# Long COVID and Incident Multimorbidity in the United States: Evidence from a Nationally Representative Longitudinal Study

**DOI:** 10.3390/healthcare14203427

**Published:** 2026-10-14

**Authors:** Ibrahim Alliu, Gulzar Shah, Subash Thapa, Emmanuel Azu, Timothy Kizza, Logan Cowan

**Affiliations:** 1 Department of Biostatistics, Epidemiology, and Environmental Health Science, Jiann-Ping Hsu College of Public Health, Georgia Southern University, Statesboro, GA 30460, USA; ia00702@georgiasouthern.edu (I.A.); st13974@georgiasouthern.edu (S.T.); ea07407@georgiasouthern.edu (E.A.); tk07429@georgiasouthern.edu (T.K.); lcowan@georgiasouthern.edu (L.C.); 2 Department of Health Policy and Community Health, Jiann-Ping Hsu College of Public Health, Georgia Southern University, Statesboro, GA 30460, USA

**Keywords:** long COVID, post-acute sequelae of SARS-CoV-2, multimorbidity, incident multimorbidity, survey-weighted logistic regression, health-related quality of life, healthcare utilization, healthcare expenditures, Medical Expenditure Panel Survey (MEPS)

## Abstract

**Background/Objectives**: Long COVID has been associated with elevated risks of individual chronic conditions, but its association with the transition to multimorbidity remains unclear. This study aims to examine whether retrospective COVID-19 status was associated with incident multimorbidity among U.S. adults. **Methods**: We analyzed Panel 24 of the Medical Expenditure Panel Survey (MEPS; 2019–2022), including 4356 individuals free of multimorbidity at baseline. COVID-19 exposure was classified as Long COVID, COVID-19-recovered, or no COVID-19. Survey-weighted logistic regression estimated associations between retrospective COVID-19 status and incident cross-domain multimorbidity, and generalized estimating equations assessed healthcare expenditures, health-related quality of life, and inpatient utilization. **Results**: Incident MM occurred in 106 participants: 17 with Long COVID, 41 COVID-19-recovered, and 48 with no COVID-19. In the primary model (4239 participants; 105 events), Long COVID was associated with higher odds of incident MM than no COVID-19 (OR 2.34; 95% CI, 1.14–4.79); the estimate for COVID-19-recovered participants was OR 1.71 (95% CI, 1.01–2.92). Fully adjusted and Firth estimates were directionally consistent. Cardiometabolic plus respiratory MM predominated (79/106). COVID-status-by-year interactions were not significant for total expenditures, out-of-pocket expenditures, physical or mental health scores, or inpatient stays. **Conclusions**: Retrospectively classified Long COVID was associated with incident cross-domain MM recorded during follow-up, but temporal ordering cannot be established, and sparse events limit precision. Replication with prospectively timed exposure and diagnosis data is needed.

## 1. Introduction

The COVID-19 pandemic, caused by severe acute respiratory syndrome coronavirus 2 (SARS-CoV-2), is one of the most consequential public health crises of recent decades. It has resulted in over 779 million confirmed infections and approximately 7 million officially recorded deaths globally as of June 2026 [1]. While much early attention focused on acute mortality and intensive care burden, a substantial proportion of survivors experience symptoms that persist for months or years after the initial infection clears. This syndrome, commonly referred to as Long COVID or post-acute sequelae of SARS-CoV-2 infection (PASC) and formally designated Post-COVID-19 Condition by the World Health Organization, has emerged as a clinically significant consequence of the pandemic [2,3]. The WHO Delphi consensus definition characterizes Post-COVID-19 Condition as symptoms that arise within three months of infection, persist for at least two additional months, and are not explained by an alternative diagnosis [2]. Estimates of Long COVID prevalence vary substantially across studies according to case definition, population, ascertainment method, vaccination status, and period of infection [4,5]. The economic consequences are substantial: Cutler [6] estimated the present value of Long COVID-related costs in the United States at approximately $3.7 trillion, though that projection carries substantial uncertainty.

Long COVID’s clinical phenotype is heterogeneous, including fatigue, cognitive impairment, dyspnea, cardiovascular manifestations including myocarditis and arrhythmia, autonomic dysfunction, and a range of neurological, immunological, and musculoskeletal complications [7]. Mechanistic investigations implicate viral persistence, immune dysregulation, reactivation of latent herpesviruses, microbiome disruption, vascular endothelial damage, and chronic neuroinflammation, among other pathways that are still being characterized [7,8]. Critically, these pathophysiological processes do not operate within a single organ system; they converge simultaneously across cardiovascular, respiratory, metabolic, neurological, and oncologic domains, creating biological conditions conducive to the accumulation of multisystem chronic disease [8,9]. Large-scale analyses using electronic health records and Veterans Affairs (VA) data have documented elevated risks of incident cardiovascular, metabolic, kidney, and neurological outcomes following COVID-19 [10,11,12]. Proposed pathways include viral persistence, immune dysregulation, endothelial injury, microvascular dysfunction, and chronic inflammation [7,8]. Evidence from other infectious lung injury contexts also demonstrates that persistent inflammation, endothelial dysfunction, and hypercoagulability can link respiratory injury to later cardiovascular and metabolic burden [13], while prior pulmonary infection has been associated with earlier onset of chronic respiratory disease and greater severity [14]. These studies do not establish a direct causal link to SARS-CoV-2, but they support the biological plausibility of chronic multisystem consequences following severe or persistent infectious injury.

Multimorbidity (MM), defined as the coexistence of two or more chronic conditions in the same individual, represents a growing public health concern both in the United States and internationally. According to recent surveillance data, more than half of U.S. adults have been diagnosed with at least one chronic condition, and a substantial proportion report two or more, collectively accounting for the majority of national healthcare expenditures and utilization [15,16]. MM affects an estimated 25–40% of adults in high-income countries and presents substantial challenges for healthcare delivery [17]. Its consequences extend well beyond the additive burden of a single condition: individuals with MM experience substantially greater healthcare utilization, higher expenditures, accelerated functional decline, and markedly reduced health-related quality of life compared with those managing a single chronic condition [17,18,19]. The transition from absent or limited chronic disease to MM represents a pivotal inflection point, a threshold at which complexity escalates, competing treatment priorities emerge, polypharmacy risk rises, and quality of life trajectories diverge sharply from those of healthier counterparts [20]. Understanding what precipitates this transition is therefore of fundamental importance to chronic disease prevention and health systems planning.

Primary care serves as the principal point of contact for most COVID-19 survivors seeking ongoing management in community settings. Despite this front-line role, much of the existing longitudinal evidence on post-COVID-19 sequelae has been generated from specialist registers, tertiary-care electronic health record networks, or VA databases, which may not adequately represent the broader community-dwelling population [10,11,12]. Nationally representative longitudinal data from community-based surveillance systems offer a complementary perspective directly relevant to how primary care teams assess post-COVID-19 chronic disease risk, identify patients approaching the MM threshold, and design follow-up care pathways for this growing population. Prior large-scale analyses using VA administrative data have documented hazard ratios ranging from approximately 1.3 to 1.7 for individual incident conditions, including cardiovascular disease, diabetes, and chronic kidney disease, among COVID-19 survivors relative to uninfected contemporaries, with elevated magnitudes among Long COVID relative to COVID-recovered individuals [10,11,12]. However, these estimates pertain to individual conditions within a predominantly older male VA population; they do not address the cross-domain multimorbidity threshold in a community-dwelling civilian sample that includes women and working-age adults. Direct numerical comparison with the present study is inappropriate because the outcomes differ: the present study examines incident multimorbidity across distinct physiological domains, whereas previous studies examined individual incident conditions. Nevertheless, the present analysis situates these findings within a broader chronic disease trajectory framework not captured in prior work.

Although individual chronic conditions that occur after COVID-19 have been well characterized, the literature has largely examined them in isolation [7,9]. Whether COVID-19 exposure, Long COVID in particular, is associated with the transition to multimorbidity across distinct physiological domains remains underexamined. A recent study using UK Biobank data found complex patterns of pre-existing multimorbidity associated with the risk of severe COVID-19 and Long COVID [21]. However, that work treated existing multimorbidity as a predictor of COVID-19 outcomes rather than the reverse. The question we address here is the inverse: whether Long COVID is associated with the transition into MM among individuals who did not have it beforehand. Prior Medical Expenditure Panel Survey (MEPS) based research established MM cluster profiles and their longitudinal associations with expenditures and quality of life in the U.S. population [22], providing a methodological foundation for assessing COVID-19-era shifts. However, nationally representative longitudinal evidence examining the association between COVID-19 exposure and subsequent transition to MM remains limited, particularly studies that also characterize healthcare expenditures, quality of life, and utilization following MM onset.

To address these gaps, we used MEPS Panel 24 (2019–2022) to examine three interrelated questions: whether retrospectively classified COVID-19 exposure status was associated with incident cross-domain MM during follow-up, with particular attention to Long COVID; whether the MM combination at onset differs by COVID-19 status; and whether post-onset trajectories of healthcare expenditures, health-related quality of life, and inpatient utilization vary across exposure groups. Together, these analyses provide nationally representative longitudinal evidence to understand Long COVID as a potential contributor to the complexity of chronic disease and for informing healthcare planning and prevention policy.

## 2. Materials and Methods

### 2.1. Study Design and Data Source

This longitudinal cohort study used MEPS Panel 24, with data from 2019–2022 from a nationally representative sample of the U.S. civilian noninstitutionalized population. MEPS employs a complex, stratified, multi-stage probability sampling design designed to produce nationally representative estimates for the U.S. civilian noninstitutionalized population [23]. Medical expenditures were adjusted for inflation to 2022 US dollars using the Gross Domestic Product (GDP) price index, consistent with AHRQ/MEPS guidance [24]. Health-related quality of life was assessed via the SF-12v2 [25], yielding Physical Component Summary (PCS) and Mental Component Summary (MCS) scores standardized to a population mean of 50 (SD = 10). All analyses were conducted in SAS 9.4 (SAS Institute Inc., Cary, NC, USA). The study used publicly available de-identified data and was exempt from Institutional Review Board (IRB) review.

### 2.2. Study Population and Eligibility

MEPS Panel 24 was selected because it is the only available MEPS panel spanning both a pre-pandemic baseline year (2019) and three consecutive COVID-19-era follow-up rounds (2020, 2021, and 2022), providing the longitudinal structure necessary to establish multimorbidity-free status prior to COVID-19 exposure and to track incident multimorbidity onset and downstream burden trajectories across the pandemic period. Participants were followed longitudinally, with information on health conditions, healthcare utilization, and expenditures collected across the study period.

**Figure 1:**
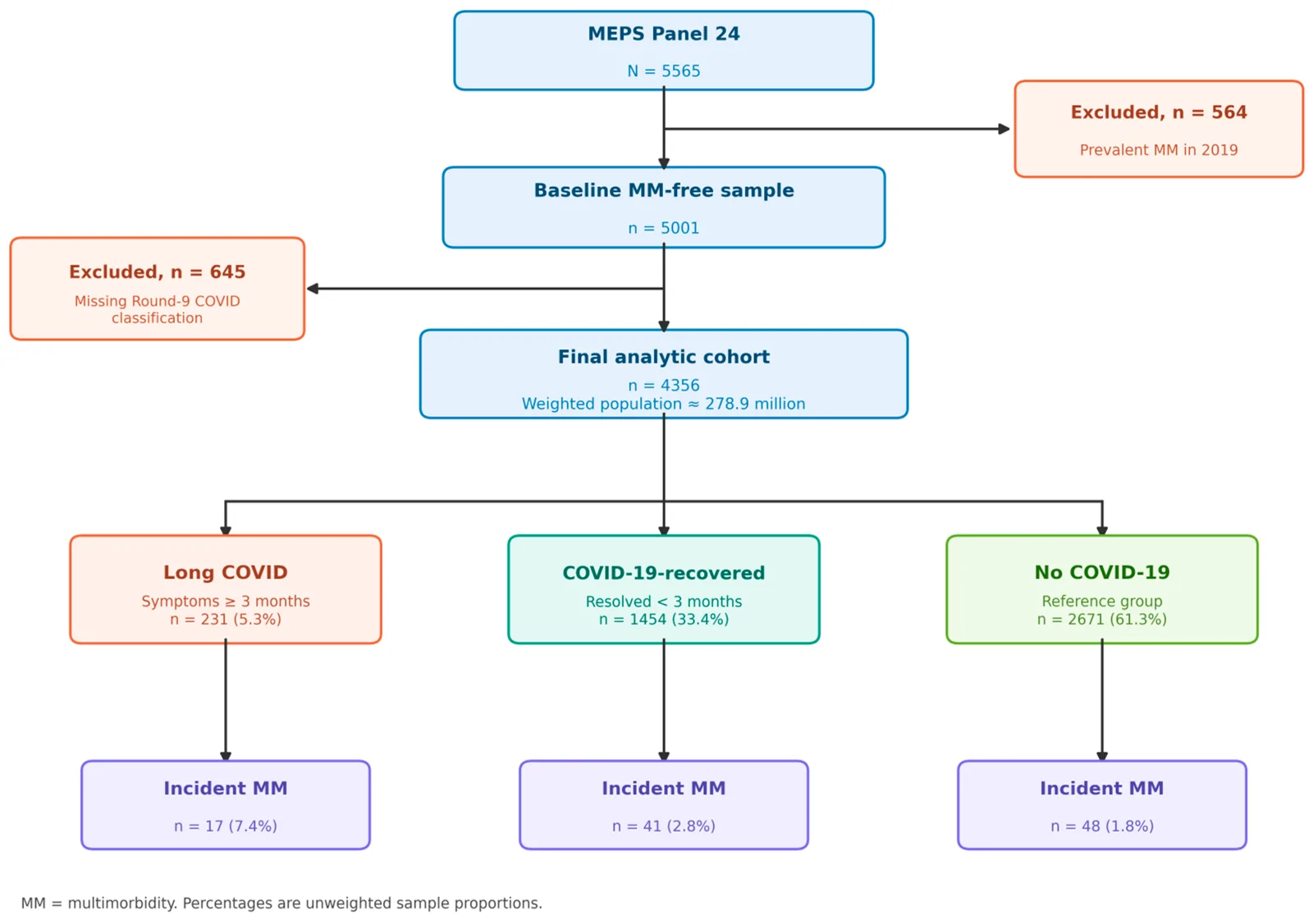
Participant flow diagram. MM = multimorbidity. Event rates are unweighted sample proportions.

Of the 5565 participants enrolled in Panel 24, 564 (9.96%) were excluded for prevalent cross-domain MM in 2019. Among the remaining 5001 participants, 645 lacked a usable Round-9 COVID classification, resulting in 4356 participants (weighted N of approximately 278.9 million; Figure 1). The analytic cohort, therefore, included participants without MM at baseline and with non-missing retrospective COVID status.

### 2.3. Exposure Classification

COVID-19 exposure was classified retrospectively using self-reported diagnosis and symptom duration into three mutually exclusive groups: Long COVID (reported COVID-19 with symptoms lasting ≥ 3 months; *n* = 231), COVID-19-recovered (reported COVID-19 with symptoms resolving within 3 months; *n* = 1454), and no reported COVID-19 (*n* = 2671; reference). Because MEPS did not capture all components of the WHO clinical definition, this measure is a self-reported survey proxy based on symptom duration rather than a clinically adjudicated diagnosis [2].

### 2.4. Outcome Definitions

#### 2.4.1. Primary Outcome: Incident MM

Incident MM was defined as the first follow-up year in which a participant had qualifying chronic conditions in two or more distinct physiological domains: cardiometabolic (diabetes, hypertension, coronary heart disease, or high cholesterol), respiratory (asthma or emphysema), and cancer (any assessed cancer site). This cross-domain definition was selected to distinguish accumulation across organ-system domains from multiple diagnoses within one domain; it is therefore narrower than the conventional definition of any two chronic conditions and should not be interpreted as directly equivalent to conventional MM prevalence estimates. Each year’s flags were derived from that year’s MEPS ever-diagnosed indicators. Conditions were not manually carried forward; persistence was represented through the cumulative diagnostic structure of those indicators. The first recorded MM was assigned to the earliest year that met the cross-domain threshold. The same annual condition-identification procedure was applied to 2020, 2021, and 2022; thus, 2022 was eligible to contribute a first-recorded MM event and was not administratively censored or excluded from incident-event ascertainment.

#### 2.4.2. Multimorbidity Cluster Taxonomy

At first recorded MM, cases were classified into four mutually exclusive domain combinations: cardiometabolic + respiratory, cancer + cardiometabolic, cancer + respiratory, or all three domains. This rule-based taxonomy followed directly from the three-domain outcome definition. Only the first three combinations were observed among incident cases; no participant first met the threshold with all three domains simultaneously.

#### 2.4.3. Secondary Outcomes: Longitudinal Healthcare Burden During and Following the Year of Recorded MM Onset

Five downstream burden outcomes were assessed across the three follow-up rounds: total healthcare expenditures (in thousands of 2022 U.S. dollars); out-of-pocket expenditures (OOP) (in thousands of 2022 U.S. dollars); physical health-related quality of life (HRQoL) as measured by the SF-12v2 Physical Component Summary score; mental health-related quality of life as measured by the SF-12v2 Mental Component Summary score; and count of inpatient hospital discharges (distinct admission episodes regardless of length of stay) during each follow-up round.

### 2.5. Statistical Analysis

Survey design variables and analytic weights were incorporated in accordance with the requirements of each analysis. The MEPS longitudinal person-level weight (LONGWT) was used for the incident-MM, expenditure, and inpatient-utilization analyses. The longitudinal Self-Administered Questionnaire weight (LSAQWT) was used for the PCS and MCS analyses because these outcomes were derived from the SF-12v2 administered through the MEPS Self-Administered Questionnaire. All tests were two-sided with α = 0.05.

#### 2.5.1. Association Between Retrospective COVID Status and Incident MM

The primary outcome was a person-level indicator of any first-recorded cross-domain MM during 2020–2022. Because the last round of COVID status did not provide the timing required for a prospective survival analysis, we used survey-weighted logistic regression rather than a discrete-time hazard model. Exponentiated coefficients are reported as odds ratios (ORs). The primary complete-case model included 4239 participants and 105 of the 106 observed events.

The primary parsimonious model included the COVID-19 group, age, baseline self-rated health, and baseline chronic condition count. Self-rated health was coded as 1 = excellent, 2 = very good, 3 = good, 4 = fair, and 5 = poor, with higher values indicating worse perceived health. The parsimonious specification was prespecified to limit sparse-data bias while retaining core demographic and baseline-health confounders. Baseline chronic-condition count was treated as a pre-exposure covariate because it was measured in 2019.

A fully adjusted sensitivity model additionally included sex, race/ethnicity, educational attainment, insurance status, and income-to-poverty category. We also fit an unweighted Firth penalized logistic model to assess sparse-event bias and an exploratory Firth model restricted to participants with no cardiometabolic, respiratory, or cancer domain at baseline. Firth estimates do not incorporate the MEPS survey design and are presented only as sensitivity analyses.

#### 2.5.2. MM Cluster Type at Onset

Among incident cases, domain combinations were summarized using unweighted counts and survey-weighted within-group percentages. Because the all-three-domain category had no cases and the remaining cells were sparse, a multinomial model was not estimable. We therefore fit an exploratory unweighted Firth logistic regression comparing cancer-involved onset (cancer + cardiometabolic or cancer + respiratory) with cardiometabolic + respiratory onset. This analysis was considered hypothesis-generating.

#### 2.5.3. Burden Trajectories During and Following Recorded MM Onset

Population-averaged longitudinal trajectories were estimated with weighted generalized estimating equations (GEE). Each model included calendar year as a categorical variable, COVID-19 group, their interaction, a time-invariant indicator identifying participants who ever developed MM, an indicator for observations at or after the first recorded MM year, years since first recorded MM, and the fully adjusted baseline covariates. Gamma-log models were used for positive total and out-of-pocket (OOP) expenditures, negative-binomial log models for inpatient stays, and normal identity-link models for PCS and MCS. An AR(1) working correlation was used for total expenditures, PCS, MCS, and inpatient stays; OOP used working independence after the AR(1) specification failed to provide a stable fit. Robust empirical standard errors were used.

The GEE analytic samples were 10,446 observations from 4042 participants for positive total expenditures; 8721 observations from 3598 participants for positive OOP expenditures; 6188 PCS observations and 6193 MCS observations from 2280 participants; and 12,669 inpatient observations from 4356 participants. A hierarchical linear growth model for log expenditures, with year treated as continuous, was used for sensitivity analysis. Exploratory effect-modification analyses used Firth models for age and insurance, a survey-weighted model for sex, and descriptive cross-tabulation for race because race-specific interaction models were not estimable.

## 3. Results

### 3.1. Study Cohort and Baseline Characteristics

Of the 5565 participants, 4356 individuals free of cross-domain MM at baseline met the inclusion criteria. After applying the longitudinal survey weight, this analytic cohort comprised approximately 278.9 million individuals. The cohort included 231 participants with Long COVID, 1454 with COVID-19-recovered status, and 2671 with no reported COVID-19 infection. During follow-up (2020–2022), 106 participants developed incident MM, corresponding to a weighted cumulative incidence of 1.98% (approximately 5.35 million individuals). Weighted incident MM was highest among participants with Long COVID (4.42%), followed by those with COVID-19-recovered status (2.54%) and those with no COVID-19 (1.32%). Unweighted sample proportions, presented in Table 1, differ from these weighted estimates because survey weights adjust for the complex stratified sampling design to produce nationally representative population-level figures; the two sets of figures address different questions and should not be directly compared.

Baseline characteristics are presented in Table 1. Compared with the COVID-19-recovered and no-COVID-19 groups, participants with Long COVID were older, had worse baseline self-rated health, and had a higher baseline chronic condition count than other groups. Distributions of race/ethnicity and poverty category varied across exposure groups, whereas sex and insurance status were broadly similar across groups. The Long COVID group had the highest proportion of non-Hispanic White participants and the lowest proportion in the high-income category.

### 3.2. Incident MM Risk

All 106 first-recorded MM events occurred in 2020 or 2021. No 2022 observations were excluded. Applying the same condition-identification algorithm to the 2022 annual variables identified no additional participants who first crossed the cross-domain multimorbidity threshold. The zero count was therefore an empirical feature of this analytic cohort rather than an administrative exclusion. The available data cannot determine whether this pattern reflects the short observation window, condition-reporting mechanics, or the underlying incidence process.

In the primary survey-weighted logistic model (4239 participants; 105 events), COVID status was associated with incident MM overall (*p* = 0.0185). Relative to no COVID-19, the odds of incident MM were higher for Long COVID (OR 2.34; 95% CI, 1.14–4.79; *p* = 0.021) and COVID-19-recovered status (OR 1.71; 95% CI, 1.01–2.92; *p* = 0.047). Baseline chronic-condition count was also associated with incident MM (OR 2.02 per condition; 95% CI, 1.47–2.78), whereas age and baseline self-rated health were not statistically significant at α = 0.05.

Results were directionally consistent in sensitivity analyses. In the fully adjusted survey-weighted model (4223 participants; 104 events), the ORs were 2.46 (95% CI, 1.09–5.57) for Long COVID and 1.66 (95% CI, 0.97–2.81) for COVID-19-recovered status. In the unweighted Firth model, corresponding ORs were 3.51 (95% CI, 1.97–6.24) and 1.48 (95% CI, 0.97–2.26). In the fully healthy baseline subset (2272 participants; 7 events), estimates were larger but highly imprecise, and the overall COVID-group test was not significant (*p* = 0.119), underscoring the sparse-event limitation. Table 2 below summarizes this result.

### 3.3. MM Cluster Type at Onset

Among the 106 cases, cardiometabolic + respiratory MM predominated (79 cases; 76.8%), followed by cancer + cardiometabolic MM (23; 19.2%) and cancer + respiratory MM (4; 4.0%). No participant first met the threshold with all three domains simultaneously. The four cancer + respiratory cases occurred only among COVID-19-exposed participants, but the cell was too small for standalone inference.

In the exploratory Firth model of 105 complete cases, there was no evidence that cancer-involved versus cardiometabolic + respiratory onset differed by COVID status (overall *p* = 0.9108). Relative to no COVID-19, the OR was 1.23 (95% CI, 0.32–4.79) for Long COVID and 1.28 (95% CI, 0.38–4.37) for COVID-19-recovered status. Older age was associated with cancer-involved onset (OR 1.09 per year; 95% CI, 1.03–1.14), whereas worse baseline self-rated health was inversely associated (OR 0.50 per category; 95% CI, 0.27–0.92). These estimates are exploratory because only 27 cases were cancer-involved.

The distribution of MM domain combinations and exploratory Firth estimates are shown in Table 3.

### 3.4. Burden Trajectories During and Following Recorded MM Onset

In the GEE models, the time-invariant indicator identifying participants who ever developed MM was associated with higher total expenditures (RR 4.06; 95% CI, 1.38–11.93) and inpatient stays (RR 4.62; 95% CI, 1.84–11.59), and with lower MCS (β −3.06; 95% CI, −5.63 to −0.48). The corresponding PCS and OOP estimates were not statistically significant. Among eventual MM cases, the at/after-onset indicator was associated with lower total expenditures relative to their pre-threshold observations (RR 0.58; 95% CI, 0.35–0.93); other post-threshold and time-since-threshold terms were imprecise. Because the model contains both a person-level case indicator and time-varying onset terms, none of these coefficients alone represents a causal effect of MM onset. 

The longitudinal associations of MM case status, recorded onset timing, and COVID-19 status with healthcare expenditures, health-related quality of life, and inpatient utilization are summarized in Table 4.

There was no evidence of a COVID-status-by-year interaction for total expenditures (*p* = 0.616), OOP expenditures (*p* = 0.652), PCS (*p* = 0.243), MCS (*p* = 0.234), or inpatient stays (*p* = 0.826). In the MCS model, Long COVID was associated with a lower adjusted score in the 2020 reference year (β = −2.37; 95% CI, −4.34 to −0.41), but the nonsignificant omnibus interaction indicates that year-specific contrasts should not be interpreted as a consistent differential trajectory. In the continuous-time hierarchical sensitivity model, the Long-COVID-by-year coefficient for log expenditures was positive (β = 0.253; 95% CI, 0.057–0.449; *p* = 0.012), indicating model-dependent evidence of steeper expenditure growth.

Exploratory interaction tests did not show statistically significant effect modification by age group (*p* = 0.138), sex (*p* = 0.965), or insurance status (*p* = 0.633). Race-specific interaction models were not estimable due to sparse cells and were therefore limited to descriptive cross-tabulation.

**Table 4:** Longitudinal associations of MM case status, recorded onset timing, and COVID status with healthcare burden trajectories.

Outcome	Ever-MM Case Indicator (95% CI)	At/After-Onset Indicator (95% CI)	Years Since Onset (95% CI)	COVID Group × Year *p*-Value
Total expenditures	RR = 4.06 (1.38–11.93)	RR = 0.58 (0.35–0.93)	RR = 0.87 (0.53–1.42)	0.616
Out-of-pocket expenditures	RR = 2.30 (0.67–7.86)	RR = 0.56 (0.16–1.97)	RR = 1.21 (0.73–1.99)	0.652
Physical HRQoL (PCS)	β = 0.82 (−1.51 to 3.14)	β = −3.80 (−7.68 to 0.08)	β = −0.83 (−3.49 to 1.84)	0.243
Mental HRQoL (MCS)	β = −3.06 (−5.63 to −0.48)	β = 1.51 (−2.09 to 5.11)	β = −0.21 (−2.47 to 2.04)	0.234
Inpatient stays	RR = 4.62 (1.84–11.59)	RR = 0.45 (0.17–1.23)	RR = 1.04 (0.65–1.68)	0.826

Note: The MM-case indicator is time-invariant and identifies participants who have developed MM; the at/after-onset indicator is time-varying; years since onset equals zero before and during the first recorded year. Log-link coefficients are exponentiated as rate ratios (RR); PCS and MCS coefficients are adjusted mean differences (β). The final column gives the omnibus COVID-group × year interaction *p*-value. Total expenditures, PCS, MCS, and inpatient stays used AR (1) working correlation; OOP used working independence. Robust empirical standard errors were used. Models adjusted for year, COVID group, their interaction, age, sex, race/ethnicity, insurance, education, poverty category, baseline self-rated health, and baseline chronic-condition count.

## 4. Discussion

### 4.1. Principal Findings

In this nationally representative longitudinal panel, retrospectively classified Long COVID was associated with higher odds of incident across-domain MM recorded during follow-up than no reported COVID-19. At the same time, the COVID-19 recovered participants also had an intermediate elevation. These findings extend prior research on individual post-COVID-19 conditions by examining a stricter threshold requiring diagnoses across distinct physiological domains. However, because COVID status was collected retrospectively in the later round, the analysis establishes an association rather than a prospective temporal or causal ordering.

The graded point estimates across no COVID-19, COVID-19-recovered, and Long COVID groups are biologically plausible but should not be overinterpreted. Persistent immune activation, endothelial injury, and prothrombotic pathways have been proposed after SARS-CoV-2 infection [7,8]. Complementary evidence from tuberculosis-associated chronic lung disease links prior infectious injury with persistent systemic inflammation, endothelial dysfunction, and hypercoagulability [13], and with earlier onset and greater severity of COPD [14]. These analogies support mechanistic plausibility but do not demonstrate that the same pathways caused the observed MM associations.

The absence of first-recorded events in 2022 was not produced by excluding that year. It arose after assigning each case to the earliest year in which cumulative annual diagnosis indicators met the two-domain threshold. Thus, 2022 contributed to longitudinal burden observations but not to any additional incident threshold crossings. Longer panels with prospectively dated diagnoses are needed to distinguish a true plateau from an artifact of the available measurement window.

### 4.2. MM Phenotype at Onset

The cross-domain outcome deliberately differs from the conventional definition of any two chronic conditions. Requiring at least two of three physiological domains increases etiologic specificity for multisystem accumulation but reduces sensitivity and event counts. Among cases, three of the four possible domain combinations were observed, with cardiometabolic + respiratory MM predominating. These patterns are descriptive and should not be generalized to conventional MM taxonomies.

Cancer + respiratory onset occurred only among COVID-19-exposed participants (*n* = 4), but this sparse cell does not support an exposure-specific inference. The Firth model likewise found no overall association between COVID status and cancer-involved versus cardiometabolic + respiratory onset.

### 4.3. Burden During and Following the Year of First Recorded MM

The burden analyses distinguish participants who eventually developed MM from the timing of their first recorded threshold. In the parameterization used, the ever-MM indicator represented differences between participants who later crossed the MM threshold and those who never developed MM when the time-varying onset terms equaled zero. The at/after-onset and years-since-onset coefficients represented additional changes among eventual MM cases and must therefore be interpreted jointly with the ever-MM coefficient. Participants who eventually developed MM had higher total expenditures and inpatient utilization and lower mental HRQoL before the additional onset-related terms were considered. Because the post-threshold coefficients were mixed and imprecise, these findings describe a high-burden case profile rather than demonstrating that the recorded MM onset caused an immediate change. For SF-12/SF-36 outcomes, meaningful-change thresholds vary by population and method: chronic disease literature often places interpretable changes near one-half standard deviation [26], while condition-specific SF-36v2 research has reported approximately four points for PCS and MCS [27]. Because the present PCS onset-related estimate was imprecise, we do not claim that it exceeded a definitive MCID.

Inpatient utilization was markedly higher among participants who had ever developed MM, but neither the at/after-onset term nor the years-since-onset term was statistically significant. This pattern is consistent with MM identifying a high-utilization group, while the short panel and uncertain timing of onset limit conclusions about progressive post-onset escalation.

By contrast, OOP expenditures were not clearly associated with incident MM onset, suggesting that the direct financial burden may depend as much on insurance design, income, and cost-sharing as on disease accumulation. Long COVID was associated with lower MCS in the 2020 reference year, but the omnibus COVID-by-year interaction was not significant; therefore, the result is best interpreted as a year-specific adjusted contrast rather than evidence of a persistently divergent mental health trajectory.

### 4.4. Methodological Considerations

An additional methodological finding was the difference between the GEE and hierarchical linear model results for total expenditures. The primary categorical-time GEE analysis did not identify a clear Long COVID-by-year interaction for any outcome, whereas the continuous-time hierarchical model analysis identified steeper growth in log expenditures over time for Long COVID participants. These models address related but not identical questions: the GEE framework emphasizes average population-level year-specific differences, while the growth-curve model evaluates differences in slope over time. Together, the two analyses suggest that any divergence in Long COVID-related expenditure may be gradual rather than concentrated within a single follow-up interval. Future studies examining the economic consequences of Long COVID may benefit from presenting both population-averaged and subject-specific trajectory models.

### 4.5. Clinical and Policy Implications

These results support cautious, hypothesis-generating implications. Primary care clinicians may consider monitoring the accumulation of chronic conditions across cardiometabolic, respiratory, and cancer domains among patients reporting prolonged post-COVID symptoms, while recognizing that this study cannot establish that COVID preceded the recorded MM threshold. The high expenditure and inpatient burden among eventual MM cases also support coordinated care for patients approaching cross-domain complexity. Clinical recommendations should await replication with prospectively timed infection, symptom, and diagnosis data.

### 4.6. International Relevance

Although drawn from U.S. data, the biological mechanisms underlying the observed association among viral immune dysregulation, endothelial dysfunction, and the accumulation of multisystem chronic disease are not health-system-specific [7,8]. MM is a recognized challenge across high-income countries, with prevalence estimates of 25–40% [17], and the present findings may be relevant to primary care systems in other settings where COVID-19 survivors are managed longitudinally. This is particularly pertinent given the ongoing contraction of dedicated Long COVID clinic infrastructure in several high-income countries. In the United Kingdom, fewer than half of NHS Long COVID services remained after the transfer of commissioning responsibility to local integrated care boards in 2025 [28]. This contraction places greater responsibility on primary care for longitudinal surveillance of chronic disease among COVID-19 survivors. However, direct generalization is limited by the U.S.-specific design, and replication in nationally representative cohorts outside the U.S. is needed before international policy recommendations can be made.

### 4.7. Limitations

This study has several limitations. First, the Round-9 COVID status was retrospective and did not provide the dates needed to establish that infection or prolonged symptoms preceded the first recorded MM. Same-year observations, therefore, cannot be temporally ordered, and the ORs should not be interpreted as hazards or causal effects. Second, self-reported COVID history and symptom duration may misclassify exposure. Differential recall is possible if participants with greater subsequent illness were more likely to remember or attribute prolonged symptoms to COVID. Residual confounding by pre-existing vulnerability, inflammatory burden, vaccination, infection severity, and healthcare access may also remain.

Differential detection is a separate concern. Participants with prolonged symptoms may have more healthcare encounters, increasing opportunities for hypertension, diabetes, respiratory disease, cancer, or other qualifying diagnoses to be detected and recorded. Such surveillance could inflate the observed association with Long-COVID even without a difference in true disease onset. Conversely, disrupted care during the pandemic may have delayed diagnosis in some groups. Visit intensity could not be modeled without risking adjustment for a consequence of illness; future claims or electronic health record studies should use prospectively measured contact frequency and diagnosis dates to examine this bias directly.

Only 106 incident MM events were observed, including 17 among participants with Long COVID. Although survey-weighted primary and fully adjusted models converged, confidence intervals were wide, and subgroup and phenotype analyses were sparse. The unweighted Firth results support directional robustness but do not account for the MEPS survey design. The cross-domain definition also yielded fewer events than the conventional any-two-condition definition, and findings may not generalize to broader MM constructs. Finally, GEE burden coefficients for person-level MM status and post-threshold timing should be interpreted jointly rather than as isolated onset effects.

## 5. Conclusions

In this nationally representative MEPS panel, retrospectively classified Long COVID was associated with higher odds of incident cross-domain MM recorded during 2020–2022 (OR 2.34; 95% CI, 1.14–4.79), with an intermediate association among COVID-19-recovered participants (OR 1.71; 95% CI, 1.01–2.92). The cardiometabolic-plus-respiratory combination was the predominant onset phenotype. Participants who eventually developed MM had higher expenditure and inpatient burden, but the COVID-specific longitudinal trajectory did not differ consistently. Because exposure timing was retrospective and events were sparse, these results are associative and hypothesis-generating. Prospective studies with dated infections and diagnoses are needed before clinical or causal conclusions are drawn. Together, these results highlight the potential relevance of chronic disease surveillance, mental health assessment, and MM-oriented management, particularly for individuals at elevated social and clinical risk.

## Figures and Tables

**Table 1:** Baseline characteristics of the analytic cohort by COVID-19 exposure status, MEPS Panel 24.

Characteristic	Total (N = 4356)	Long COVID (*n* = 231)	COVID-19-Recovered (*n* = 1454)	No COVID-19 (*n* = 2671)
Age, mean (SE)	38.67 (0.57)	46.35 (1.38)	41.32 (0.68)	36.29 (0.75)
Sex, %				
Male	49.65	41.36	48.86	50.83
Female	50.35	58.64	51.14	49.17
Race/Ethnicity, %				
Non-Hispanic White	57.52	72.09	62.97	52.97
Non-Hispanic Black	12.94	6.73	11.24	14.51
Hispanic	19.75	17.23	16.76	21.79
Non-Hispanic Asian	6.26	2.67	6.00	6.72
Non-Hispanic Other/Multiple	3.52	1.28	3.03	4.02
Insurance Status, %				
Insured	89.12	86.02	90.35	88.61
Uninsured	10.88	13.98	9.65	11.39
Poverty category, %				
Poor/Negative/Near Poor	16.69	16.13	11.39	20.13
Low/Middle Income	39.69	47.26	37.97	40.11
High Income	43.62	36.60	50.65	39.76
Baseline Health Indicators				
Chronic Condition Count, Mean (SE)	0.41 (0.01)	0.60 (0.07)	0.41 (0.02)	0.39 (0.02)
Self-Rated Health, Mean (SE)	2.07 (0.02)	2.43 (0.10)	2.07 (0.04)	2.03 (0.03)

Note: Sample sizes (*n*) are unweighted counts. Percentages and continuous-variable estimates are survey-weighted to represent the U.S. noninstitutionalized civilian population. Continuous variables are presented as weighted mean (SE), and categorical variables as weighted percentages.

**Table 2:** Survey-weighted logistic association between retrospective COVID-19 status and incident cross-domain multimorbidity, MEPS Panel 24 (2019–2022).

Model	Predictor	Events	Estimate	95% CI
Primary survey-weighted model	Long COVID vs. No COVID-19	17	OR 2.34	1.14–4.79
	COVID-19-recovered vs. No COVID-19	41	OR 1.71	1.01–2.92
	Age, per year	—	OR 1.01	0.99–1.03
	Baseline self-rated health, per category	—	OR 1.26	1.00–1.60
	Baseline chronic-condition count, per condition	—	OR 2.02	1.47–2.78
	Long COVID vs. No COVID-19	17	OR 2.46	1.09–5.57
Fully adjusted survey-weighted	COVID-19-recovered vs. No COVID-19	41	OR 1.66	0.97–2.81
	Long COVID vs. No COVID-19	17	OR 3.51	1.97–6.24
Unweighted Firth	COVID-19-recovered vs. No COVID-19	41	OR 1.48	0.97–2.26

Note: The primary and fully adjusted models are survey-weighted logistic regressions and report odds ratios. The primary model included 4239 participants and 105 events; the fully adjusted model included 4223 participants and 104 events. The Firth model was unweighted and included 4239 participants. No COVID-19 was the reference group. Event counts are the 106 unweighted events observed in the full analytic cohort. OR = odds ratio; CI = confidence interval.

**Table 3:** Domain combination at onset and exploratory binary model.

A. Incident MM cluster type at onset				
**Domain Combination**	**Long COVID (** ***n*** ** = 17)**	**COVID-19-Recovered (** ***n*** ** = 41)**	**No COVID-19 (** ***n*** ** = 48)**	**Total (** ***n*** ** = 106)**
Cardiometabolic + respiratory	11 (62.2%)	32 (82.4%)	36 (74.4%)	79 (76.8%)
Cancer + cardiometabolic	4 (26.4%)	7 (11.8%)	12 (25.6%)	23 (19.2%)
Cancer + respiratory	2 (11.5%)	2 (5.8%)	0 (0.0%)	4 (4.0%)
All three domains	0	0	0	0
Note: Counts are unweighted sample frequencies; percentages are survey-weighted within-group estimates.				
B. Exploratory Firth logistic model: cancer-involved vs cardiometabolic + respiratory onset				
**Predictor**			**Estimate**	**95% CI**
Long COVID vs No COVID-19			OR 1.23	0.32–4.79
COVID-19-recovered vs No COVID-19			OR 1.28	0.38–4.37
Age, per year			OR 1.09	1.03–1.14
Baseline self-rated health, per category			OR 0.50	0.27–0.92
Note: Unweighted Firth penalized logistic regression based on 105 complete incident cases. Reference COVID group: no COVID-19. Reference outcome: cardiometabolic + respiratory onset. Cancer-involved combines cancer + cardiometabolic and cancer + respiratory. Survey weights were not applied. OR = odds ratio; CI = confidence interval.				

## Data Availability

Publicly available datasets were analyzed in this study. The data are available from the Agency for Healthcare Research and Quality (AHRQ) Medical Expenditure Panel Survey (MEPS) Panel 24 Longitudinal Public Use File (HC-245) at https://meps.ahrq.gov/mepsweb/data_stats/download_data_files_detail.jsp?cboPufNumber=HC-245 (accessed on 27 July 2026). No new datasets were generated during the current study.

## Abbreviations

The following abbreviations are used in this manuscript:

AHRQ	Agency for Healthcare Research and Quality
AR(1)	Autoregressive Correlation Structure of Order 1
CI	Confidence Interval
COVID-19	Coronavirus Disease 2019
GEE	Generalized Estimating Equations
HRQoL	Health-Related Quality of Life
Long COVID	Post-COVID-19 Condition
MCS	Mental Component Summary
MEPS	Medical Expenditure Panel Survey
MM	Multimorbidity
OOP	Out-of-Pocket
OR	Odds Ratio
PASC	Post-Acute Sequelae of SARS-CoV-2 Infection
PCS	Physical Component Summary
RR	Rate Ratio
SARS-CoV-2	Severe Acute Respiratory Syndrome Coronavirus 2
SAS	Statistical Analysis System
SD	Standard Deviation
SE	Standard Error
SF-12v2	12-Item Short Form Health Survey Version 2
U.S.	United States
WHO	World Health Organization
